# Extranucleosomal DNA enhances the activity of the LSD1/CoREST histone demethylase complex

**DOI:** 10.1093/nar/gkv388

**Published:** 2015-04-27

**Authors:** Sang-Ah Kim, Nilanjana Chatterjee, Matthew J. Jennings, Blaine Bartholomew, Song Tan

**Affiliations:** 1 Center for Eukaryotic Gene Regulation, Department of Biochemistry & Molecular Biology, 108 Althouse Laboratory, The Pennsylvania State University, University Park, PA 16802-1014, USA; 2 UT MD Anderson Cancer Center, Department of Epigenetics and Molecular Carcinogenesis, 1808 Park Road 1C, Smithville, TX 78957, USA

## Abstract

The promoter regions of active genes in the eukaryotic genome typically contain nucleosomes post-translationally modified with a trimethyl mark on histone H3 lysine 4 (H3K4), while transcriptional enhancers are marked with monomethylated H3K4. The flavin-dependent monoamine oxidase LSD1 (lysine-specific demethylase 1, also known as KDM1) demethylates mono- and dimethylated H3K4 in peptide substrates, but requires the corepressor protein, CoREST, to demethylate nucleosome substrates. The molecular basis for how the LSD1/CoREST complex interacts with its physiological nucleosome substrate remains largely unknown. We examine here the role of extranucleosomal DNA beyond the nucleosome core particle for LSD1/CoREST function. Our studies of LSD1/CoREST's enzyme activity and nucleosome binding show that extranucleosomal DNA dramatically enhances the activity of LSD1/CoREST, and that LSD1/CoREST binds to the nucleosome as a 1:1 complex. Our photocrosslinking experiments further indicate both LSD1 and CoREST subunits are in close contact with DNA around the nucleosome dyad as well as extranucleosomal DNA. Our results suggest that the LSD1/CoREST interacts with extranucleosomal DNA when it productively engages its nucleosome substrate.

## INTRODUCTION

The assembly of DNA around histone proteins into the nucleosome is used to package the eukaryotic genome into the nucleus (1). The histone proteins do not simply act as passive spools to wrap DNA, but instead play active roles in the regulation of gene expression (2,3). For example, post-translational modifications of histones including methylation, acetylation, phosphorylation and ubiquitylation mediate transcriptional responses to intra- and extra-cellular signals and thus constitute important gene regulatory mechanisms (4).

Lysine methylation is a key post-translational histone modification because it is associated with both transcriptional activation and repression, and because many of the enzymes responsible for lysine methylation and demethylation are linked with human cancers (5–7). Each lysine residue can be mono-, di- or trimethylated, and the specific form of methylation at specific lysine residues can be functionally important. For example, the promoter regions of transcriptionally active genes are marked with histone H3 Lys4 (K4) trimethylation, while enhancers are marked with H3K4 monomethylation (8–10). The Jumanji domain protein, JARID1B, demethylates H3K4me2 and H3K4me3, while the LSD1 protein demethylates H3K4me1 and H3K4me2 (11). LSD1's catalytic activity is contained in its FAD-dependent amine oxidase domain, and this domain is sufficient to bind to the N-terminal tail of histone H3 (12–16). While the LSD1 protein is able to catalyze demethylation of methylated H3K4 peptides, it is not able to catalyze the demethylation on methylated H3K4 incorporated into nucleosomes (12,17). The nucleosome specific enzymatic activity requires an additional protein, CoREST, which heterodimerizes with LSD1. The LSD1/CoREST complex forms an elongated structure with the LSD1 amine oxidase and SWIRM domains on one end, a CoREST SANT domain on the other, and a helical linker in between formed by both LSD1 and CoREST (12).

The catalytic activity and H3 peptide binding properties of LSD1 and the LSD1/CoREST complex are relatively well-understood due to extensive biochemical and structural studies (11,18). In contrast, we possess little information regarding LSD1/CoREST's interactions with the nucleosome, its physiological substrate. In this study, we examine LSD1/CoREST's catalytic and binding activities on nucleosome substrates. We find that the LSD1/CoREST complex is catalytically more active and binds more tightly to nucleosomes containing extranucleosomal DNA. Furthermore, our photocrosslinking studies suggest that LSD1/CoREST may bind to nucleosomal DNA around the nucleosomal dyad and on extranucleosomal DNA one to two helical turns beyond the end of the nucleosome core particle.

## MATERIALS AND METHODS

### Protein expression and purification

The coding regions for human LSD1 and CoREST were amplified from HeLa cDNA. Hexahistidine-tagged human LSD1Δ1 = LSD1(171–852) and CoRESTΔ1 = CoREST(286–482) were expressed using the pST44-polycistronic expression vector (19) in BL21(DE3)pLysS cells at 23°C. The coexpressed LSD1Δ1/CoRESTΔ1 complex (referred in the text as LSD1/CoREST) was purified by Talon (Clontech) cobalt affinity chromatography before TEV protease digestion to remove the hexahistidine tag, followed by SourceQ (GE Healthcare) anion-exchange chromatography.

Recombinant *Xenopus* core histones and nucleosome core particles were prepared as described previously (20). All nucleosomes were purified by SourceQ anion-exchange high performance liquid chromatography.

### Fluorescent labeling

Histones engineered to contain site-specific cysteine mutations were fluorescently labeled by incubating in 7 M guanidine–HCl, 20 mM Tris–HCl pH 7.5 and 0.25 mM Tris(2-carboxyethyl)phosphine (TCEP) with a 2-fold molar excess of Oregon Green-488 maleimide dye (LifeTech) overnight at 4°C. Histone H3 mutants were created in the H3(C110A) background to ensure unique labeling at the engineered position. Typical labeling efficiencies of 10–30% were observed.

### LSD1/CoREST histone demethylase assay

Nucleosome substrates containing a methyl lysine analog of dimethylated H3K4 were prepared by chemically modifying a site-directed mutagenized cysteine residue to mimic methylated H3K4 (21). In particular, recombinant H3K4C protein was alkylated with 2-chloro-*N*,*N*-dimethylethylamine hydrochloride (Aldrich) to produce *N*,*N*-dimethylated aminoethylcysteine modified H3 previously shown to be recognized by H3K4 specific antibodies (21). This alkylation of histone H3 was confirmed by LC-MS analysis (data not shown). The H3K_C_4me2 protein was reconstituted into nucleosomes as described above.

LSD1/ CoREST activity on nucleosomes with various DNA lengths was measured by a peroxidase-coupled assay, which monitors hydrogen peroxide production (22). Amplex^®^ UltraRed (Invitrogen), which is colorless and nonfluorescent, produces highly fluorescent resorufin upon oxidation (23). The 100 μl reactions in 96-well microplate (Greiner) were initiated by the addition of 10 μl of 500 nM LSD1/CoREST solution to reaction mixtures (90 μl) consisting of 20 mM HEPES-NaOH (pH 7.5), 50 mM NaCl, 10 μM Amplex^®^ UltraRed and 0.76 μM horseradish peroxidase. Fluorescence changes were monitored with excitation at 530 nm and emission at 590 nm using a BioTek Synergy H4 Hybrid Multi-Mode Microplate Reader. The initial velocity values were fitted to the Michaelis–Menten equation using GraphPad Prism software to determine *V*_max_ and *k*_cat_ values. At 50, 150 and 200 nM of the limiting nucleosome substrates, these initial velocity values were determined using fluorescence data up to when 26%, 25% and 13% of 147 bp nucleosomes were consumed respectively. The equivalent percentages were 38%, 31% and 25% for 157 bp nucleosomes; 13%, 19% and 17% for 165 bp nucleosomes and 57%, 34% and 32% for 185 bp nucleosomes.

### HI-FI nucleosome binding assay

LSD1/CoREST interaction with nucleosomes with different DNA lengths was measured by the HI-FI (high-throughput interactions by fluorescence intensity) procedure as described (24,25). Site-specific Oregon Green 488 labeled nucleosomes (0.2–5 nM) were titrated with 5 nM to 10 μM LSD1/CoREST complex in 20 mM Tris–HCl (pH 7.6), 50 mM NaCl, 5 mM DTT, 5% glycerol, 0.01% NP40, 0.01% 3-CHAPS ([(3-cholamidopropyl)dimethylammonio]-1-propanesulfonate), 100 μg/ml BSA (bovine serum albumin) and 2 mM EDTA (ethylenediaminetetraacetic acid), and incubated at room temperature for 15 min. Fluorescence was monitored with excitation at 488 nm and emission at 526 nm. Assays were performed at least three times and the dissociation constants determined using the single binding isotherm equation 11.1 in Winkler *et al*. Initial experiments indicated Hill coefficients of 1.18, 1.08, 1.01 and 0.98 for 147, 157, 181 and 207 bp nucleosomes, respectively, and we therefore used a Hill coefficient of 1.0 for subsequent analysis.

We selected nucleosome positions H3 K27C and H3 A21C based on the largest fluorescence changes using near saturating concentrations of LSD1/CoREST at 50, 75 and 100 mM NaCl (Supplementary Figure S1). We observed reduced fluorescence change with increasing extranucleosomal DNA from 147 to 157 to 181 bp for nucleosomes labeled on H2A T10C and H2B K20C, but the reverse for nucleosomes labeled on H3 A21C and H3 K27C. These findings are consistent with the probes on H2A T10C and H2B K20C detecting nonspecific binding of LSD1/CoREST to the nucleosome, binding that is reduced at higher stringency conditions of increased NaCl concentrations.

### HI-FI stoichiometry assay

Stoichiometric measurements were performed with 805 nM of 181 bp nucleosome labeled on H3 A21C at 100 mM NaCl. The nucleosomes were titrated with LSD1/CoREST at a ratio of 0.1:1 to 5:1 [LSD1/CoREST]:[nucleosome]. The two linear phases of the plot of normalized fluorescence change versus concentration ratio were each fit with a line. The binding stoichiometry corresponds to the concentration ratio at the intersection between the two linear phases of the plot (24,25).

### Photocrosslinking assay

Synthesis of photoreactive phosphorothioate DNA probes: A series of 36 photoreactive phosphorothioate DNA probes were synthesized as described earlier (26) to scan LSD1/CoREST interactions with 147 bp of nucleosomal DNA and approximately 35 bp of linker DNA from the edge of the nucleosome. Of these 36 probes, 10 were positioned in the extranucleosomal DNA and the remaining 26 were positioned every 5 bp in the nucleosomal DNA with the photocrosslinker either facing towards or away from the histone octamer. Eighteen to twenty-mer oligonucleotide primers complementary to these 36 positions in the extranucleosomal and nucleosomal DNA were commercially synthesized with a phosphorothioate moiety incorporated between the third and fourth nucleotides from the 5′ end. The oligonucleotide primers were incubated at 25°C for 1 h with APB (*p*-azido phenacyl bromide) in the dark to attach the photocrosslinker to the phosphorothioate moiety through a 7 Å tether. Chemically synthesized phosphorothioate oligonucleotides exist as a racemic mixture and therefore allowed the conjugated photocrosslinker to project towards both major and minor grooves of DNA.

The template DNA for photoreactive probe synthesis was prepared by digestion of the plasmid p159-1-G4 27-Xba1 with NdeI, incorporating biotinylated nucleotides, Bio-14-dATP (Invitrogen) and Bio-11-dUTP (Enzo) with Klenow exo^−^ DNA polymerase, and HindIII digestion followed by immobilizing the biotinylated DNA on Dynabeads M-280 Streptavidin (Invitrogen). Non-biotinylated DNA strand was removed by denaturation with 0.1 M sodium hydroxide. The bead bound single stranded template DNA was dephosphorylated with calf intestinal alkaline phosphatase and employed for photoreactive probe synthesis.

The APB modified oligonucleotides generated earlier were 5′-end radiolabeled and annealed to the single stranded template. The annealed oligonucleotides were extended by T4 DNA Polymerase in presence of all four dNTPs, and also T4 DNA ligase and ATP added simultaneously to the reaction to seal the nicks after extension. Two hundred and twenty-three base pairs photoreactive phosphorothioate DNA probes were released from the beads by digestion with EcoRI restriction enzyme.

Synthesis of photoreactive nucleosomes: Nucleosomes (34N42) containing the 601 positioning sequence (27) flanked by 34 and 42 bp of extranucleosomal DNA were assembled separately in the dark with the 36 different photoreactive DNA probes, salmon sperm carrier DNA and recombinant *Xenopus laevis* histone octamer at 30°C by stepwise salt dilution from 2 M to 280 mM NaCl and analyzed by 4% (35:1 acrylamide to bisacrylamide) native polyacrylamide gel electrophoresis.

Site specific DNA photoaffinity crosslinking: Each of the 36 different 34N42 photoreactive and radiolabeled nucleosomes (100 nM) were separately incubated with or without LSD1/CoREST (860 nM) at 30°C for 30 min in a 12.5 μl reaction containing 20 mM HEPES–NaOH (pH 7.8), 3 mM MgCl2, 6% (v/v) glycerol, 95 mM NaCl and 0.1 μg/μl BSA. Nucleosome binding by LSD1/ CoREST was assessed by loading 2 μl of the binding reaction on a 4% (79:1 acrylamide to bis-acrylamide) native polyacrylamide gel. Remaining binding reactions were then subjected to 2 min UV irradiation (310 nm, 2.65 mW/cm^2^, at the distance of 8 cm) to crosslink protein subunits in close vicinity of the photocrosslinker with radiolabeled DNA followed by digestion with 4.6 units of DNase I (Ambion) at 30°C for 15 min. SDS was added to a final concentration of 0.4% and samples heated at 90°C for 3 min. Heating in the presence of SDS released DNA from the histone octamer. The crosslinked protein–DNA samples were cooled on ice, brought to room temperature, supplemented with 1 mM zinc acetate and further digested with 20 units of S1 nuclease (USB) at 30°C for 15 min. Digestion with DNase I and S1 nuclease removed excess DNA to reduce altering the mobility of crosslinked protein subunits on SDS-PAGE. The nuclease digested samples were analyzed by 4–20% gradient Tris–glycine SDS-PAGE followed by western transfer on a nitrocellulose membrane at 80 V for 6 h at 4°C. The LSD1 & CoREST subunits crosslinked at different positions in the nucleosomal and extranucleosomal DNA were visualized by autoradiography by exposing the membrane to a screen and scanning it using a phosphorimager (Fuji).

## RESULTS

### Steady state kinetics activity suggest role for extranucleosomal DNA

**Figure 1.**
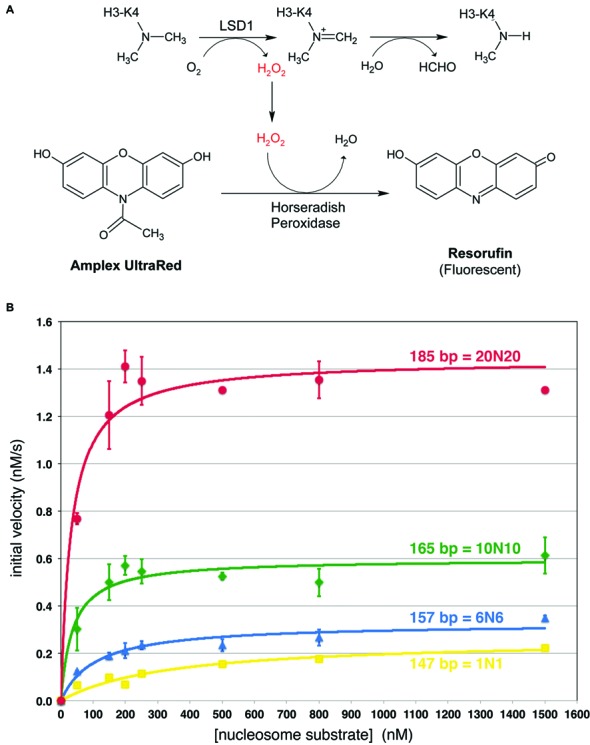
Kinetic analysis of LSD1/CoREST demethylase on nucleosome substrates. (**A**) Schematic of Amplex Ultrared based fluorescence LSD1/CoREST demethylase assay. The hydrogen peroxide generated in the histone demethylase assay drives the horseradish peroxidase catalyzed conversion of Amplex UltraRed to the highly fluorescent resorufin molecule. (**B**) Michaelis–Menten analysis of LSD1/CoREST demethylase activity on nucleosomes with symmetrical extranucleosomal DNA. Kinetic data for 147 bp (1N1), 157 bp (6N6), 165 bp (10N10) and 185 bp (20N20) are shown in yellow, blue, green and red, respectively. Each data point was measured at least three times.

Our studies of LSD1/CoREST lysine demethylase activity on nucleosome substrates (MW > 200 kDa) required a more sensitive peroxidase assay than used previously with peptide substrates (MW = 2–3 kDa) (14,22) due to the nearly 100 fold difference in substrate molecular weight. We therefore employed the reagent Amplex UltraRed which is converted to the highly fluorescent resorufin dependent on hydrogen peroxide produced during lysine demethylation (28) (Figure 1a). We reconstituted recombinant nucleosome core particles containing a methyl lysine analog of dimethylated H3 Lys 4 (H3K_C_4) and 147 bp of the Widom 601 nucleosome positioning sequence. Since the 601 nucleosome core particle encompasses 145 bp of DNA (29,30), this 147 bp 601 nucleosome core particle includes one additional base pair on either end of the central core particle, i.e. the 147 bp sequence constitutes a 1N1 positioning sequence where N represents the central 145 bp 601 nucleosome core particle sequence. Our data from this assay appear to follow Michaelis-Menten kinetics. We find that LSD1/CoREST demethylates H3K_c_4me2 147 bp nucleosomes with a *k*_cat_ of 0.315 min^−1^ and a *K*_m_ of 339 nM (Figure 1b, Table 1). This represents a slower turnover than LSD1 protein (without CoREST) on a 21 amino acid H3K4me2 peptide (*k*_cat_ = 8.1 min^−1^) or LSD1/CoREST on a 21 amino acid H3K4me1 peptide (*k*_cat_ = 7.4 min^−1^), and a smaller *K*_m_ than LSD1 or LSD1/CoREST (4.2 μM for LSD1 with H3K4me2 peptide, 5.1 μM for LSD1/CoREST with H3K4me1 peptide) (15,22). We note that these peptide studies were performed using methylated lysine residues, whereas our nucleosomes studies employed methylated aminoethylcysteine analogs of lysine.

**Table 1.** Kinetic constants for LSD1 complex using nucleosome substrates containing different extranucleosomal DNA lengths

DNA length	DNA format	*k*_cat_ (min^−1^)	*K*_m_ (M)	*k*_cat_/*K*_m_ (M^−1^ s^−1^)
147	1 + 145 + 1	0.315 ± 0.022	339 ± 60 x 10^−9^	1.6 × 10^4^
157	6 + 145 + 6	0.392 ± 0.019	107 ± 20 x 10^−9^	6.1 × 10^4^
165	10 + 145 + 10	0.717 ± 0.031	36.8 ± 10 x 10^−9^	3.3 × 10^5^
185	20 + 145 + 20	1.73 ± 0.049	32.3 ± 6.5 x 10^−9^	8.9 × 10^5^

We considered the comparatively lower *k*_cat_ of LSD1/CoREST on the 147 bp nucleosome substrate and wondered if the activity of the enzyme would be affected by lengthening the extranucleosomal DNA. We prepared equivalent nucleosomes containing the same preparation of histone H3K4me2 methyl lysine analog but now with 157 bp (6N6), 165 bp (10N10) and 185 bp (20N20) nucleosomal DNA. In each case, the extranucleosomal DNA was extended symmetrically from the central 145 bp 601 sequence. We observe a slight increase in *k*_cat_ to 0.392 min^−1^ for the 157 bp nucleosome substrate, and an apparent three fold decrease of *K*_m_ (107 nM) (Figure 1b, Table 1). Unfortunately, we are unable to reliably measure the activity using LSD1/CoREST enzyme concentrations or nucleosome substrate concentrations below 50 nM due to poor signal to noise ratios. Since steady state kinetics may not apply under these conditions due to significant concentrations of enzyme–substrate complex present, we are likely overestimating the concentration of unbound substrate. Consequently this measured *K*_m_ probably represents an upper limit. More significant changes in *k*_cat_ were observed using nucleosomes with even longer DNA fragments. The 165 bp (10N10) nucleosomes which extend the DNA by 9 bp on each side compared to the 147 bp substrate increase *k*_cat_ almost 2-fold (0.717 min^−1^ versus 0.315 min^−1^) while the 185 bp (20N20) nucleosomes increase *k*_cat_ more than 5-fold over the 147 bp nucleosomes (1.73 min^−1^) (Figure 1b, Table 1). The longer DNA also further decrease the *K*_m_ to less than 40 nM for both the 165 and 185 bp nucleosomes, with the same caveat for the 157 bp nucleosomes that these values may be overestimates.

The concomitant increase in *k*_cat_ and decrease in *K*_m_ results in a higher *k*_cat_/*K*_m_ specificity constant for nucleosomes with longer extranucleosomal DNA. Our results suggest specificity constants of 1.6 x 10^4^, 6.1 × 10^4^, >3 × 10^5^ and >9 x 10^5^ M^−1^ s^−1^ for nucleosomes containing 147, 157, 165 and 185 bp, respectively (Table 1). It is worth noting that because the same preparation of histone H3 modified with the K4 methyl lysine analog was used to reconstitute each nucleosome, it is unlikely that these differences in the specific constants reflect differences in amount of methyl lysine modification. We also note that since the *K*_m_ determined for the 157, 165 and 185 bp substrates may be overestimates, the specificity constants for these substrates could be even higher. Thus, it appears that LSD1/CoREST is significantly more efficient on nucleosomes containing extranucleosomal DNA.

### LSD1/CoREST binds more tightly to nucleosomes with extranucleosomal DNA

Since our steady state kinetics studies indicated that LSD1/CoREST was more efficient on nucleosomes with extranucleosomal DNA, we next compared LSD1/CoREST's ability to bind to nucleosomes with different lengths of extranucleosomal DNA. We employed the HI-FI nucleosome binding assay developed by Luger *et al*. (24,25) to measure the affinity of LSD1/CoREST for its nucleosome target. In this assay, one detects the quenching of a fluorescent dye installed on the nucleosome due to the binding of the chromatin protein to the nucleosome. This requires the dye to be located on a nucleosome position that is close enough to the chromatin protein to be detected without interfering with binding.

**Figure 2.**
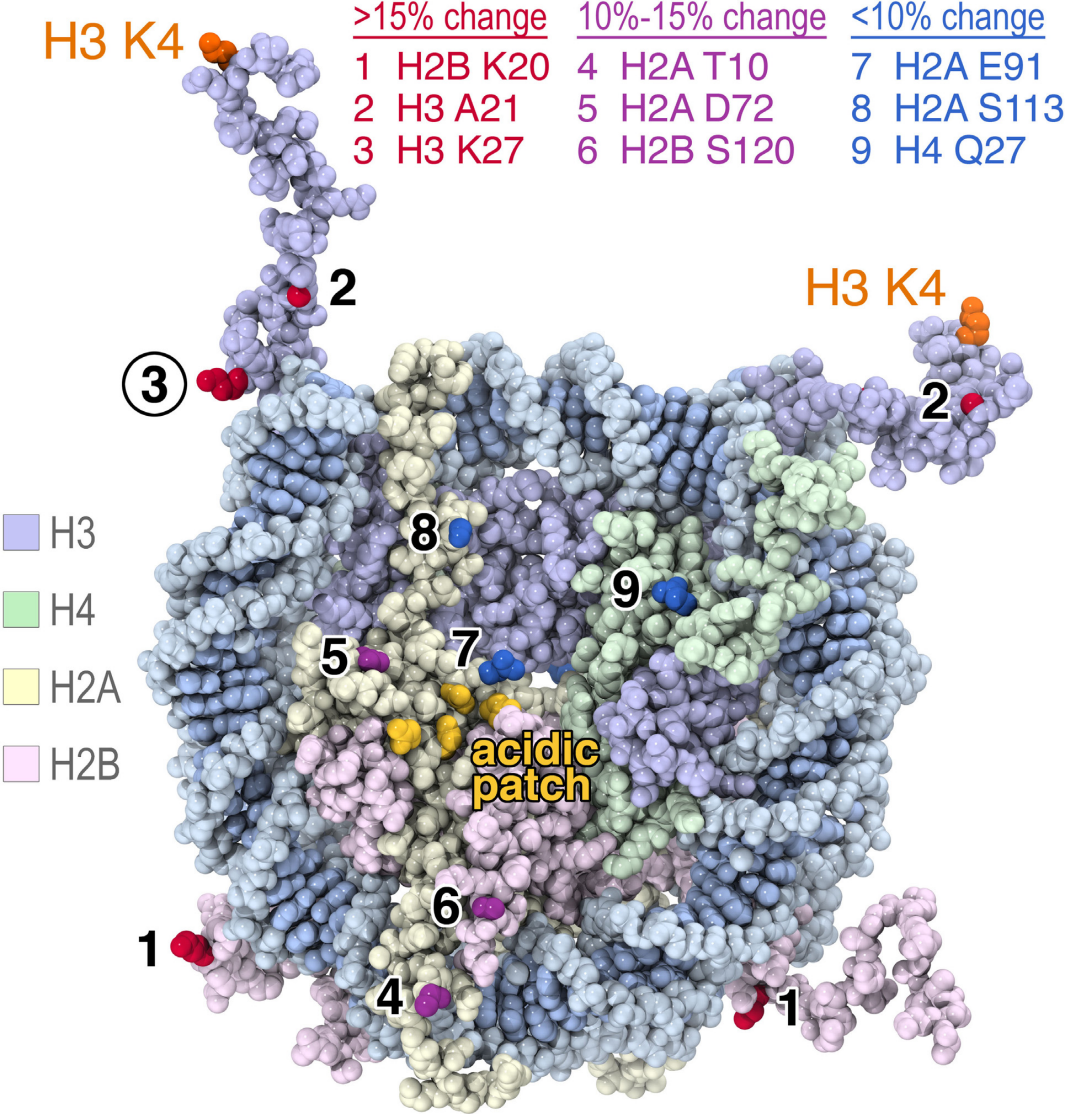
Fluorescence positions on the nucleosome affected by LSD1/CoREST binding. Oregon Green 488 was conjugated site-specifically to histones engineered with unique cysteines, reconstituted with appropriate histones and Widom 601 147 bp DNA and used in fluorescence quenching nucleosome binding experiments. The results are mapped on the space filling representation of the 1.9 Å crystal structure of the nucleosome core particle (PDB code 1KX5). Nucleosomes labeled on H2B K20, H3 A21 and H3 K27 showed >15% fluorescence quenching upon LSD1/CoREST binding (side chains shown in red), nucleosome labeled on H2A T10, H2A D72 and H2B S120 produced 10–15% fluorescence quenching (purple residues) while nucleosomes labeled on H2A E91, H2A S113 and H4 Q27 produced less than 10% fluorescence quenching (blue residues). The four H2A residues in the nucleosome acidic patch, E61, E64, D90 and E92, are shown in yellow. Figure prepared using PyMOL molecular graphics software (37).

To scout for suitable nucleosome positions, we prepared histones containing Cys substitution at nine histone positions across the nucleosome surface: on H2A Thr10, Asp72, Glu91, Ser113; on H2B Lys20, Ser120, H3 Ala21, Lys27; and on H4 Gln27 (Figure 2). The Oregon Green 488 maleimide was conjugated to the individual Cys mutant histone protein, and each fluorescently labeled histone was then reconstituted into recombinant nucleosomes. We tested these fluorescently labeled nucleosomes in binding experiments with LSD1/CoREST complex at 50 mM NaCl and found little or no (<10%) change in fluorescence for nucleosomes labeled on H2A E91C, H2A S113C and H4 Q27C (data not shown), suggesting that these positions are either located far away from LSD1/CoREST or alternatively, that fluorescent labeling at these positions inhibits binding of LSD1/CoREST to the nucleosome. Nucleosomes labeled on H2A T10C, H2A D72C and H2B S120C produced measurable fluorescence changes (between 10 and 15%) when mixed with LSD1/CoREST complex. However, incubation of LSD1/CoREST with nucleosomes labeled at H2B K20C, H3 A21C and H3 K27C resulted in large changes (>15%) in fluorescence. The fluorescent changes for nucleosomes labeled at H3 A21C and H3 K27C were particularly dramatic, with changes of 30–60% detected (data not shown), more than the minimum of 10% recommended for quantitative studies (25).

**Figure 3.**
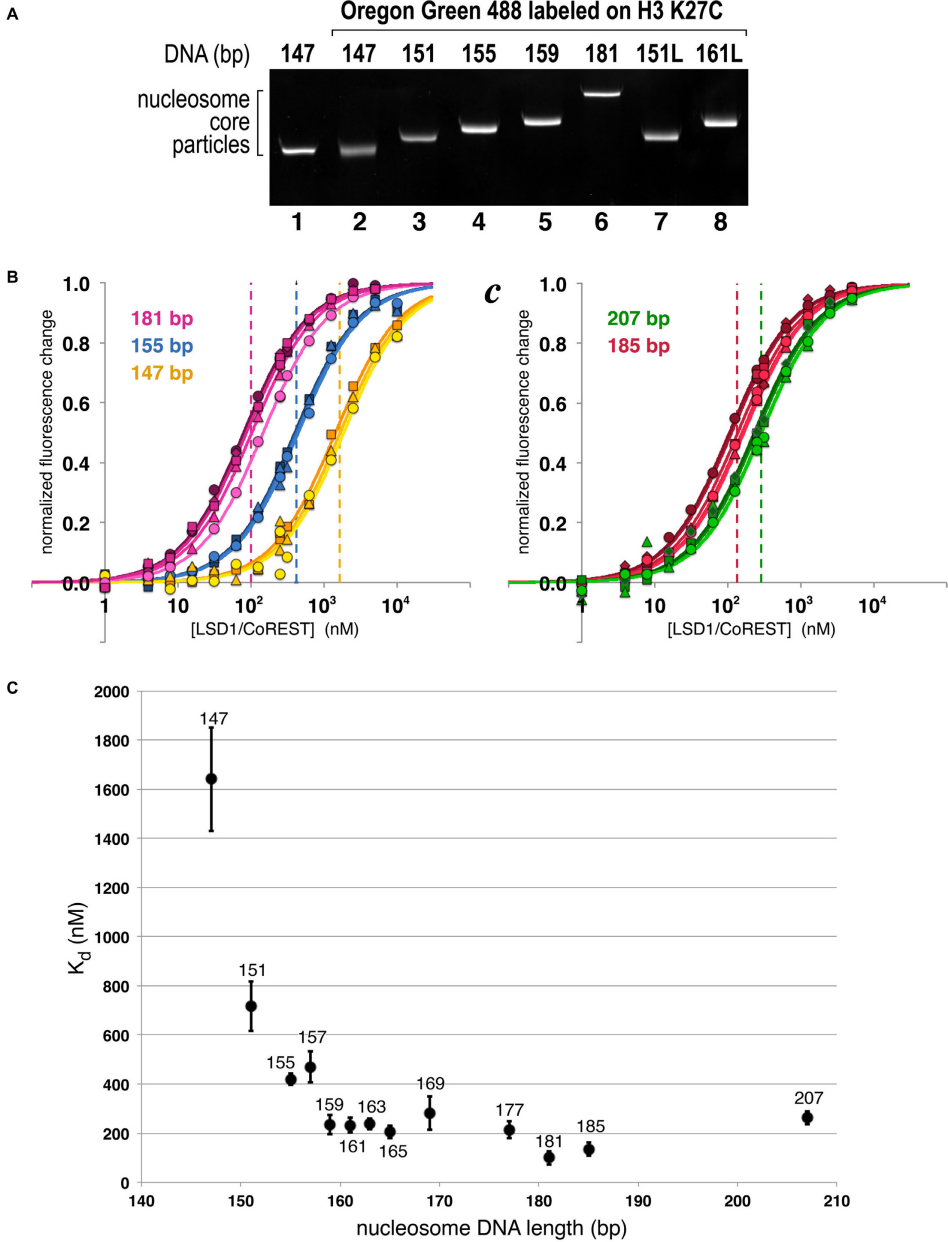
Extranucleosomal DNA increases nucleosome binding affinity of LSD1/CoREST. (**A**) Purified recombinant nucleosome core particles used for nucleosome binding assay as separated on native 10% acrylamide gel and visualized by ethidium bromide staining. (**B**) Binding curves for LSD1/CoREST interacting with 147 (yellow), 155 (blue) and 181 bp (pink) nucleosomes fluorescently labeled on H3 K27C. Three or more binding curves for individual experiments were performed for each nucleosome, with typical standard deviations of 15% of less for the calculated dissociation constants. The dashed vertical lines correspond to the calculated dissociation constants for each respective binding curve. (**C**) Equivalent binding curves for LSD1/CoREST interacting with 185 (red) and 207 bp (green) nucleosomes. LSD1/CoREST binds more weakly to the 207 bp nucleosomes compared to the 185 bp nucleosomes despite the longer extranucleosomal DNA. (**D**) Summary of dissociation constants for 13 nucleosomes containing 147–207 bp symmetrically positioned DNA. The standard deviations from three or more measurements are shown as vertical bars for each data point.

We selected the H3 K27C position to use in our LSD1/CoREST nucleosome binding assay. Nucleosomes labeled on H3 K27C (Figure 3a) were titrated with 5 nM to 10 μM LSD1/CoREST, and the normalized fluorescence change plotted as a function of the LSD1/CoREST concentration. We performed each titration at least three times in separate experiments using fresh dilutions of the LSD1/CoREST complex. The data in Figure 3 and Table 2 demonstrate that we are able to obtain reproducible results with typical standard deviations of 15% or less.

**Table 2.** Dissociation constants for LSD1 complex binding to nucleosomes with different length extranucleosomal DNA at 50 mM NaCl

Label position	DNA length	Histones	DNA format	*K*_d_ (nM)	Relative to 147 bp
H3 K27C	**147**	WT	1 + 145 + 1	1641 ± 211	1.0
	**151**	WT	3 + 145 + 3	716 ± 100	2.3
	**155**	WT	5 + 145 + 5	419 ± 23	3.9
	157	WT	6 + 145 + 6	470 ± 64	3.5
	**159**	WT	7 + 145 + 7	236 ± 40	7.0
	161	WT	8 + 145 + 8	232 ± 29	7.1
	163	WT	9 + 145 + 9	237 ± 22	6.9
	165	WT	10 + 145 + 10	205 ± 26	8.0
	169	WT	12 + 145 + 12	282 ± 68	5.8
	177	WT	16 + 145 + 16	214 ± 35	7.7
	**181**	WT	18 + 145 + 18	100 ± 27	16.2
	185	WT	20 + 145 + 20	135 ± 27	12.1
	**207**	WT	31 + 145 + 31	263 ± 27	6.2
	151L	WT	5 + 145 + 1	718 ± 31	2.3
	161L	WT	15 + 145 + 1	406 ± 52	4.0
	177L	WT	31 + 145 + 1	769 ± 174	2.1
	177	Acidic patch mutation	16 + 145 + 16	98 ± 27	16.7
H3 A21C	147	WT	1 + 145 + 1	81.0 ± 9.0	1.0
	157	WT	6 + 145 + 6	17.6 ± 1.9	4.6
	181	WT	18 + 145 + 18	3.8 ± 1.1	21.4
	207	WT	31 + 145 + 31	3.0 ± 1.3	27.2

We find that LSD1/CoREST binds 147 bp nucleosomes with a dissociation constant of 1.64 μM (Figure 3b, Table 2). To analyze the effect of extranucleosomal DNA on LSD1/CoREST binding, we reconstituted nucleosomes fluorescently labeled on H3 K27C using nucleosomal DNA lengths from 151 to 207 bp, each extended symmetrically about the central 601 bp sequence (Figure 3a). Extending the 147 bp DNA fragment by just 2 bp on either side to 151 bp (3N3) results in a *K*_d_ of 716 nM or ∼2× greater affinity (Figure 3b, Table 2). Adding an additional 2 bp on either side produces a 155 bp nucleosomal DNA (5N5) and almost a 2-fold decrease in *K*_d_ to 419 nM, while the 159 bp DNA (7N7) further reduced the dissociation constant to 236 nM or almost seven times greater affinity compared to the 147 bp nucleosomes. Extending the DNA from 159 to 177 bp did not produce significant changes in the dissociation constant. We did observe a reproducible reduction in the *K*_d_ with 181 bp nucleosomes to 100 ± 27 nM (Figure 3b, Table 2), while LSD1/CoREST bound 185 and 207 bp nucleosomes with dissociation constants of 135 ± 27 and 263 ± 27 nM, respectively (Figure 3c, d, Table 2).

We validated our observation that extranucleosomal DNA increases the affinity of LSD1/CoREST using nucleosomes labeled on H3 A21C, the other labeling position that produced large changes in fluorescence upon LSD1/CoREST binding. We observe the same trend of increased binding affinity from 147 to 157 bp to 181 bp, but not the decreased binding affinity with 207 bp nucleosomes (Table 2 and Supplementary Figure S2). Surprisingly, the measured apparent dissociation constants using nucleosomes labeled on H3 A21C were ∼20-fold lower than using nucleosomes labeled on H3 K27C. The simplest explanation is that the fluorescent probe installed on H3 K27C inhibits LSD1/CoREST binding, or the probe installed on H3 A21C enhances LSD1/CoREST binding, or some combination of the two occurs. While the precise dissociation constants presented here should therefore be viewed with some skepticism, consistent trends in our results establish an important role of extranucleosomal DNA for LSD1/CoREST binding to the nucleosome.

### LSD1/CoREST appears to engage extranucleosomal DNA on both sides of the nucleosome

**Figure 4.**
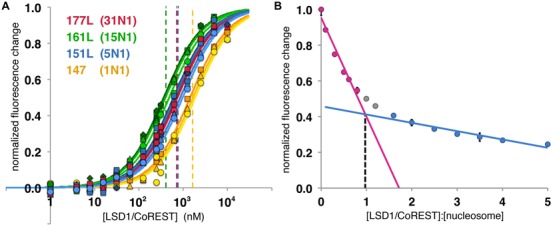
Effect of extranucleosomal DNA on one side of the nucleosome on binding affinity to LSD1/CoREST and stoichiometry of LSD1/CoREST binding to the nucleosome. (**A**) Binding curves for LSD1/CoREST interacting with 147 (1N1, yellow), 151L (5N1, blue), 161L (15N1, green) and 177L (31N1, red) nucleosomes fluorescently labeled on H3 K27C. Three or more binding curves for individual experiments were performed for each nucleosome. The dashed vertical lines correspond to the calculated dissociation constants for each respective binding curve. (**B**) Stoichiometry plot for LSD1/CoREST binding to 181 bp nucleosomes fluorescently labeled on H3 A21C. LSD1/CoREST was titrated with a fixed concentration of nucleosomes and the normalized fluorescence change plotted as a function of ratio of [LSD1/CoREST] to [nucleosomes]. The plot indicates a stiochiometry ratio 0.972 LSD1/CoREST molecules per nucleosome.

To examine the possibility that LSD1/CoREST interacts with extranucleosomal DNA on only one side of the nucleosome, we assayed binding to nucleosomes containing extranucleosomal DNA extensions on one side of our standard 147 bp nucleosomes (Figure 4a, Table 2). The 151L nucleosomes (5N1) extends the DNA by 4 bp on the left side and decreases the *K*_d_ from 1641 to 718 nM, similar to the 716 nM dissociation constant observed for the symmetrical 151 bp nucleosome of the same DNA length. However, adding an additional 10 bp on the left side (161L = 15N1) further decreases the *K*_d_ to 406 nM versus 232 nM for the symmetrical 161 bp nucleosomes. These results suggest that LSD1/CoREST requires extranucleosomal DNA on both sides of the nucleosome for maximal binding.

Surprisingly, extending the nucleosomal DNA an additional 16 bp in the 177L (31N1) nucleosomes increased the *K*_d_ to 769 nM, or slightly weaker affinity than the 151L (5N1) nucleosomes despite a substantially longer extranucleosomal DNA (Figure 4a, Table 2). This decreased affinity mirrors the result for the symmetrical 207 bp nucleosomes (31N31) which bound LSD1/CoREST less tightly than the symmetrical 181 or 185 bp nucleosomes. A possible interpretation of these results is discussed below.

### Stoichiometry of LSD1/CoREST binding to the nucleosome

We have determined the stoichiometry of LSD1/CoREST binding to the nucleosome using the HI-FI stoichiometry assay described by Winkler *et al*. (25). LSD1/CoREST was titrated with the high affinity 181 bp symmetrically positioned nucleosomes fluorescently labeled on H3 A21C. The plot of normalized fluorescence change as a function of the LSD1/CoREST:nucleosome concentration ratio shows data that can be fit with two lines which intersect at a binding ratio of 0.972 or ∼1.0 (Figure 4b). We therefore conclude that LSD1/CoREST binds to the 181 bp nucleosome with a 1:1 stoichiometry. This is consistent with the recent determination that LSD1/CoREST binds to 146 bp nucleosome core particles as a 1:1 complex (31).

### Photocrosslinking studies of LSD1/CoREST on the nucleosome

**Figure 5.**
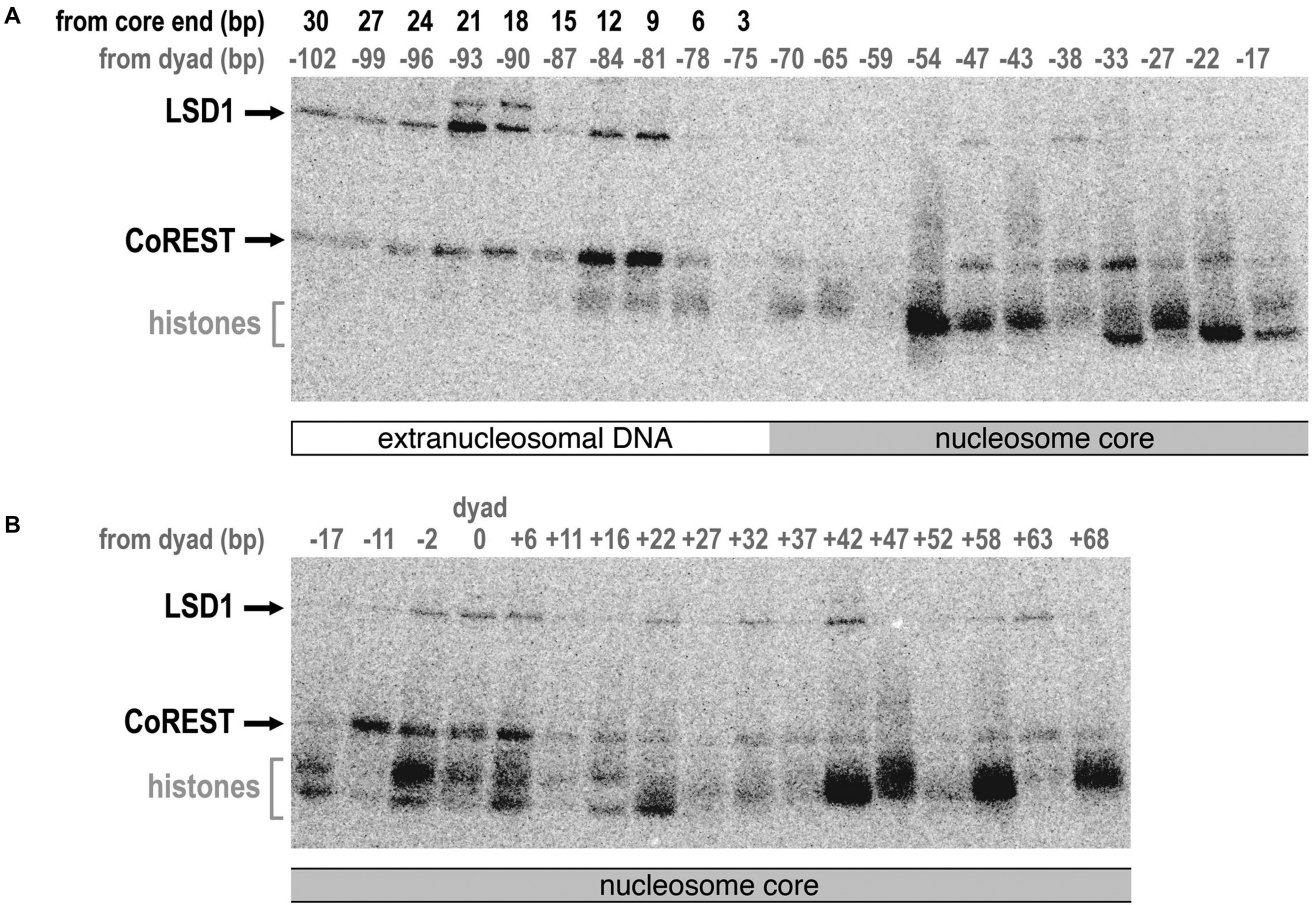
Photocrosslinking of LSD1/CoREST to site-specifically labeled nucleosomes. (**A**) Autoradiograph of SDS-PAGE gel shows LSD1 and CoREST subunits crosslinked to nucleosomes site-specifically modified with an aryl azide photoreactive group from −102 to −17 bp from the nucleosome dyad. The base pair positions from the dyad are shown in gray, while the base pair positions from the 145 bp nucleosome core particle end are show in black above the autoradiograph. The migration of LSD1, CoREST and histone proteins are shown on the left. (**B**) Equivalent autoradiograph for photocrosslinking of LSD1/CoREST to nucleosomes site-specifically modified with photoreactive group from −17 to +68 bp from the nucleosome dyad.

The steady state kinetics and nucleosome binding assays suggest that LSD1/CoREST interacts with extranucleosomal DNA. We next performed photocrosslinking studies to further investigate these findings and to gain insight into which subunit of the LSD1/CoREST complex is in close proximity to nucleosomal DNA. In particular, we used a library of 38 nucleosomes each containing a photoactivatable crosslinking group incorporated along the DNA phosphate backbone close to a radiolabel (32). Each nucleosome contained the same 221 bp DNA sequence with the 145 bp 601 nucleosome positioning sequence located between a 34 bp extension on one side, and 42 bp on the other (34N42). The library consists of nucleosomes with an aryl azide photoreactive group incorporated approximately every 5 bp within the central 145 bp and every 3 bp in the extranucleosomal DNA. The LSD1/CoREST complex was incubated with each nucleosome, exposed to UV irradiation to crosslink the appropriate protein subunit to the DNA, treated with nucleases to remove excess DNA, separated by SDS-PAGE and visualized by autoradiography (Figure 5). As expected, crosslinks to the histone proteins are detected at many positions within the nucleosome core. In addition, crosslinks to both LSD1 and CoREST proteins are detected at three distinct positions. Strong crosslinks to CoREST and weaker crosslinks to LSD1 are observed near the nucleosome dyad, while some crosslinks are observed ∼30–40 bp on one side of the dyad corresponding to the opposite edge of the nucleosome core particle relative to the dyad.

**Figure 6.**
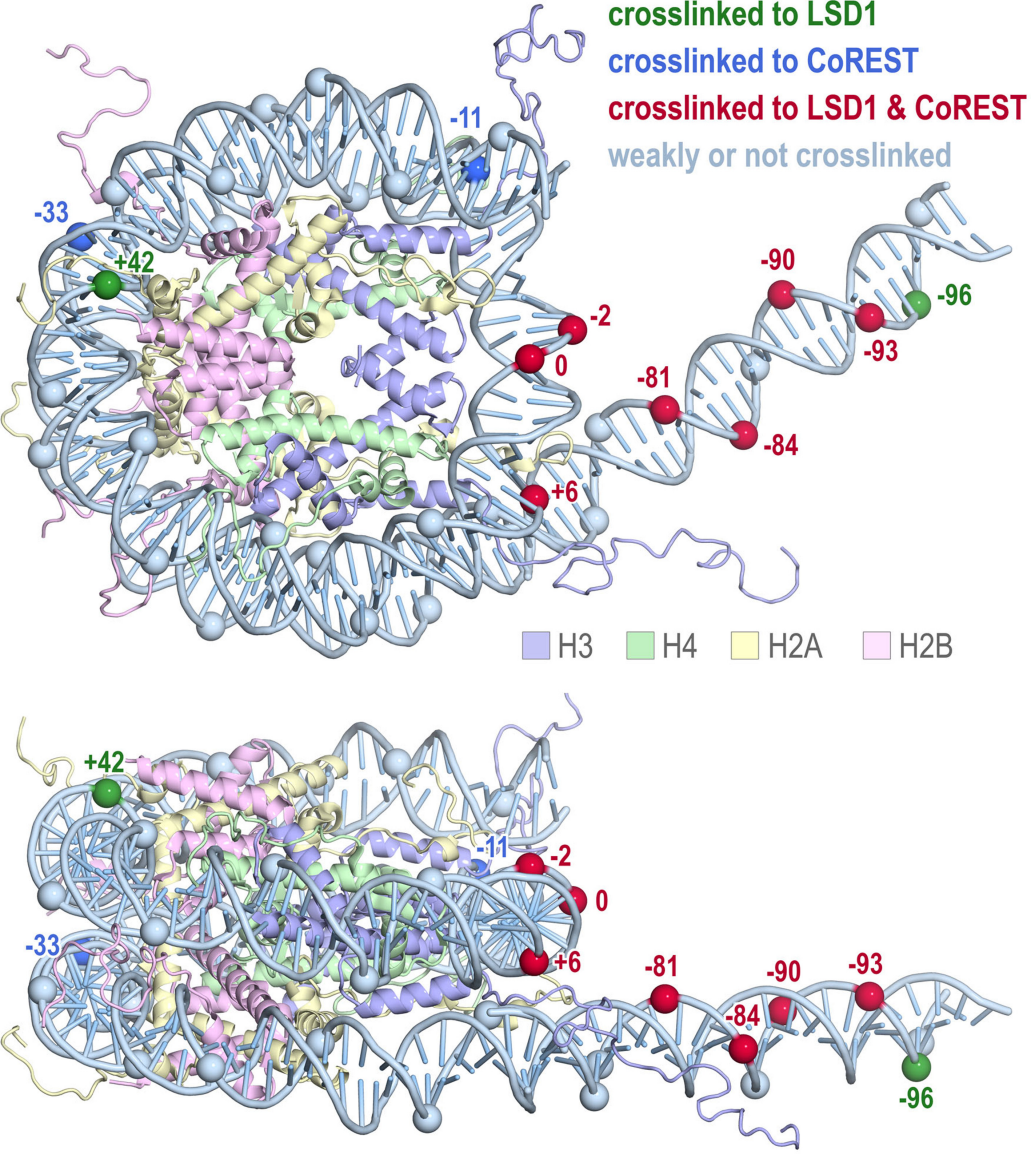
Photocrosslinking results mapped onto the structure of the nucleosome core particle. The molecule shown contains the histone octamer from the 1.9 Å structure of the nucleosome core particle to show the extent of the histone tails (PDB code 1KX5), the DNA from the 2.5 Å structure of the 601 nucleosome core particle to show the 145 bp nucleosomal DNA (PDB code 3LZ0), and 31 bp of modeled B-form extranucleosomal DNA. Positions along the phosphate backbone crosslinked to LSD1, CoREST and both LSD1/CoREST are shown in green, blue and red and labeled with the base pair position from the nucleosome dyad, while positions with little or no crosslinking are shown in light blue. Figure prepared using PyMOL molecular graphics software (37).

The strongest crosslinks to both LSD1 and CoREST occur on the extranucleosomal DNA. CoREST crosslinks strongly to extranucleosomal DNA approximately one helical turn from the end of the nucleosome core particle and more weakly one turn beyond. LSD1 also crosslinks to these locations, but with reversed intensities (stronger to DNA two helical turns from nucleosome core particle end, weaker to DNA one turn from core particle end). When modeled on the three dimensional structure of the nucleosome, the LSD1 and CoREST crosslinks to the nucleosomal dyad and extranucleosomal DNA align to create a potential contiguous interaction surface or face (Figure 6).

### LSD1/CoREST does not employ an arginine anchor to interact with the nucleosome acidic patch

Crystal structures of three chromatin proteins (the RCC1 chromatin factor (29), the Sir3 silencing protein (33), the Polycomb PRC1 ubiquitylation complex (34)) and two peptides (the herpesviral LANA peptide (35) and a CENP-C centromeric peptide (36)) bound to the nucleosome have been determined to date. All five structures share a common ‘arginine anchor’ motif where the chromatin protein or peptide inserts an arginine side chain into an acidic patch on the histone dimer surface of the nucleosome formed by H2A residues E61, E64, D90 and possibly E92 (locations shown in Figure 2). Given this common use of the arginine anchor as a nucleosome recognition motif, we asked if LSD1/CoREST would also interact with this nucleosome acidic patch. We prepared a recombinant nucleosome containing the H2A E61A, E64A, D90A, E92A quadruple mutation, H3 fluorescently labeled on K27C, and 177 bp of 601 DNA (16N16), and used this nucleosome in our binding assay. This acidic patch quadruple mutation eliminated binding and catalytic activity of the PRC1 ubiquitylation complex with the nucleosome (34). We find that LSD1/CoREST binds the 177 bp nucleosome containing H2A E61A, E64A, D90A, E92A mutations with 98 ± 27 nM dissociation constant, similar to the equivalent non-mutated nucleosome with 181 bp DNA and ∼2-fold higher affinity than to equivalent non-mutated nucleosomes with 177 bp DNA (Table 2). This result indicates that LSD1/CoREST does not make critical interactions to the nucleosome acidic patch, nor does it employ an arginine anchor. In fact, it appears that the acidic patch partially inhibits binding of LSD1/CoREST to the nucleosome.

## DISCUSSION

Our results show that extranucleosomal DNA increases both the catalytic activity of the LSD1/CoREST complex on nucleosome substrates as well as nucleosome substrate binding affinity. These findings indicate that LSD1/CoREST interacts with extranucleosomal DNA beyond the nucleosome core particle and that these interactions positively influence the histone demethylase activity of the complex.

Our enzymatic, binding and crosslinking studies suggest that LSD1/CoREST binds productively to the nucleosome near the nucleosome dyad, as well as to approximately 20 bp of extranucleosomal DNA extending from the end of the nucleosome core particle. We observe maximal binding to 181 bp symmetric nucleosomes, containing 18 bp extranucleosomal DNA on each end. We also detect crosslinking of LSD1 and CoREST to extranucleosomal DNA ∼10 and 20 bp from the nucleosome core particle, as well as to DNA around the nucleosome dyad. Altogether these results highlight the role of extranucleosomal DNA in directing LSD1/CoREST activity. Furthermore, we identified only three histone positions where LSD1/CoREST binding significantly quenched fluorescence of the probes incorporated into nucleosomes (H2B K20, H3 A21 and H3 K27). In contrast, modest or no change in fluorescence was detected at the six other histone positions examined, many on the histone face of the nucleosome. This suggests that LSD1/CoREST does not bind to the histone face of the nucleosome, a conclusion supported by our finding that the histone dimer quadruple acidic patch mutation does not adversely affect LSD1/CoREST binding to the nucleosome. Rather, it appears likely that LSD1/CoREST interacts with the nucleosome primarily through interactions with nucleosomal DNA including extranucleosomal DNA.

If LSD1/CoREST binds nucleosomes productively predominantly via interactions with nucleosome DNA around the nucleosome dyad and with extranucleosomal DNA, why did we detect fluorescence changes using nucleosomes labeled at the H2A T10C and at H2B K20C positions located away from the nucleosome dyad (Figure 2) and why did we detect crosslinking to nucleosomal DNA at −33 and +42 bp from the nucleosome dyad? We suspect that these interactions reflect nonspecific binding of LSD1/CoREST to nucleosomal DNA because these interactions are weakened by increased salt concentrations even in the presence of extranucleosomal DNA (Supplementary Figure S1). In the absence of extranucleosomal DNA required for the preferred binding mode of LSD1/CoREST on the nucleosome, LSD1/CoREST might bind nonspecifically to nucleosomal DNA producing a modest change in fluorescence at the H2A T10C label close to nucleosomal DNA. Increasing the salt concentration in the absence of extranucleosomal DNA reduces this nonspecific binding. The decrease is further amplified in the presence of extranucleosomal DNA as the preferred LSD1/CoREST binding mode, to DNA around the nucleosome dyad and extending from the nucleosome core particle, is favored. This interpretation is also consistent with a recent study which shows that LSD1/CoREST binds DNA nonspecifically apparently through the CoREST SANT domain (31).

We propose that LSD1/CoREST binds as a 1:1 complex to the nucleosome core particle as well as to extranucleosomal DNA to position the LSD1 amine oxidase domain for catalysis with the histone H3 N-terminal tail. This would explain our nucleosome binding and crosslinking results as well as our enzymatic assays showing that extranucleosomal DNA increase catalytic activity of the LSD1/CoREST complex. Pilotta *et al*. have recently proposed that LSD1/CoREST may scan nucleosomal DNA via nonspecific DNA binding (31), and we extend this concept to suggest additional interactions with extranucleosomal DNA may position LSD1/CoREST for appropriate binding of histone H3 tail and subsequent catalysis.

Our nucleosome binding studies showed increased binding affinity when the nucleosome core particle is symmetrically extended with extranucleosomal DNA up to 181 bp (18N18), but extensions to 185 bp (20N20) and 207 bp (31N31) led to an unexpected decrease in binding affinity when nucleosome labeled on H3 K27C were used. Similarly, lengthening nucleosomes on one side from 147 bp (1N1) to 151L (5N1) to 161L (15N1) increased binding affinity, but the further extension to 177L (31N1) decreased binding affinity back to the level of 151L. This decrease in binding affinity occurred despite close contact with extranucleosomal DNA observed in our photocrosslinking experiments 10–20 bp from the central 145 bp nucleosome core particle. Several explanations could account for these observations. It is possible that LSD1/CoREST bends or distorts extranucleosomal DNA beyond 181 bp of symmetrically extended nucleosome DNA and that such DNA distortions expend binding energy. It is also possible that the LSD1/CoREST protein complex undergoes conformational changes to engage undistorted extranucleosomal DNA. Another possible explanation is that a second LSD1/CoREST could bind to the extranucleosomal DNA in such a way that competes with LSD1/CoREST detected by the installed fluorescent probe. We currently cannot distinguish between these possibilities and we do not have an explanation for the essentially unchanged apparent dissociation constant for 207 versus 181 bp nucleosomes when nucleosomes labeled on H3 A21C were used.

Our biochemical analysis provides only very low resolution information for how LSD1/CoREST binds to its nucleosome substrate. Nevertheless, it seems likely that both LSD1 and CoREST subunits interact with the nucleosomal DNA near the nucleosomal dyad as well as with extranucleosomal DNA, presumably to position LSD1's catalytic domain to bind to and act on the H3 N-terminal tail. Further investigations, particularly structural studies, should help determine precisely how the various domains and regions of the LSD1 and CoREST proteins interact with elements of the nucleosome to achieve appropriate positioning for catalysis.

## SUPPLEMENTARY DATA

Supplementary Data are available at NAR Online.

SUPPLEMENTARY DATA
