# Supplementary material for: Extranucleosomal DNA enhances the activity of the LSD1/CoREST histone demethylase complex
Source: Nucleic Acids Res. 2015 Apr 27;43(10):4868–80. doi: 10.1093/nar/gkv388 (PMC4446439; doi:10.1093/nar/gkv388)
Supplement: SUPPLEMENTARY DATA [file supp_43_10_4868__index.html]

Extranucleosomal DNA enhances the activity of the LSD1/CoREST histone demethylase complex — Extranucleosomal DNA enhances the activity of the LSD1/CoREST histone demethylase complex — SUPPLEMENTARY DATA 

# Extranucleosomal DNA enhances the activity of the LSD1/CoREST histone demethylase complex

## SUPPLEMENTARY DATA

**Files in this Data Supplement:**

- SUPPLEMENTARY DATA
