# Supplementary material for: Extranucleosomal DNA enhances the activity of the LSD1/CoREST histone demethylase complex
Source: Nucleic Acids Res. 2015 Apr 27;43(10):4868–80. doi: 10.1093/nar/gkv388 (PMC4446439; doi:10.1093/nar/gkv388)
Supplement: SUPPLEMENTARY DATA [file supp_gkv388_nar-03533-x-2014-File008.pdf]

Supplementary Material

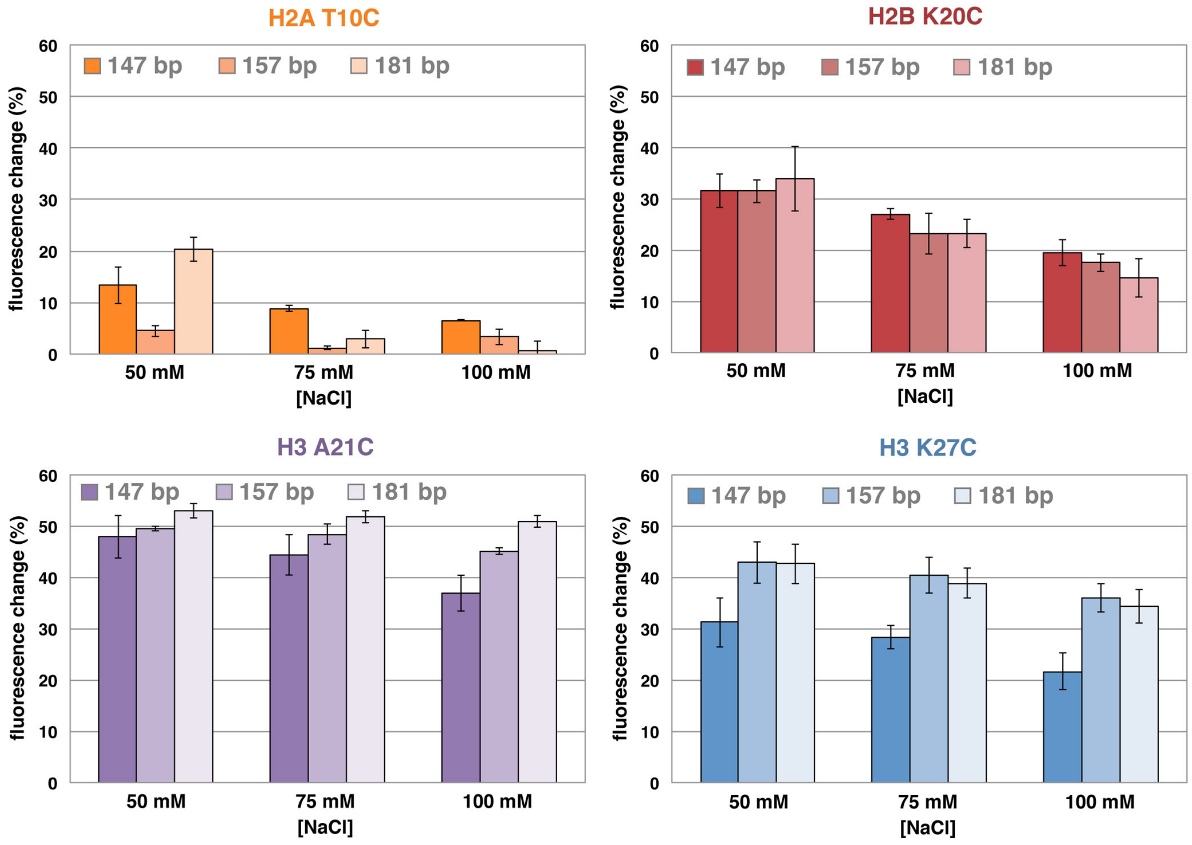

Supplementary Figure 1: Fluorescence change for 147 bp , 157 bp and 181 bp nucleosomes labeled on H2A T10C, H2B K20C, H3 A21C and H3 K27C in the presence of near saturating concentrations of LSD1/CoREST at 50, 75 and 100 mM NaCl.

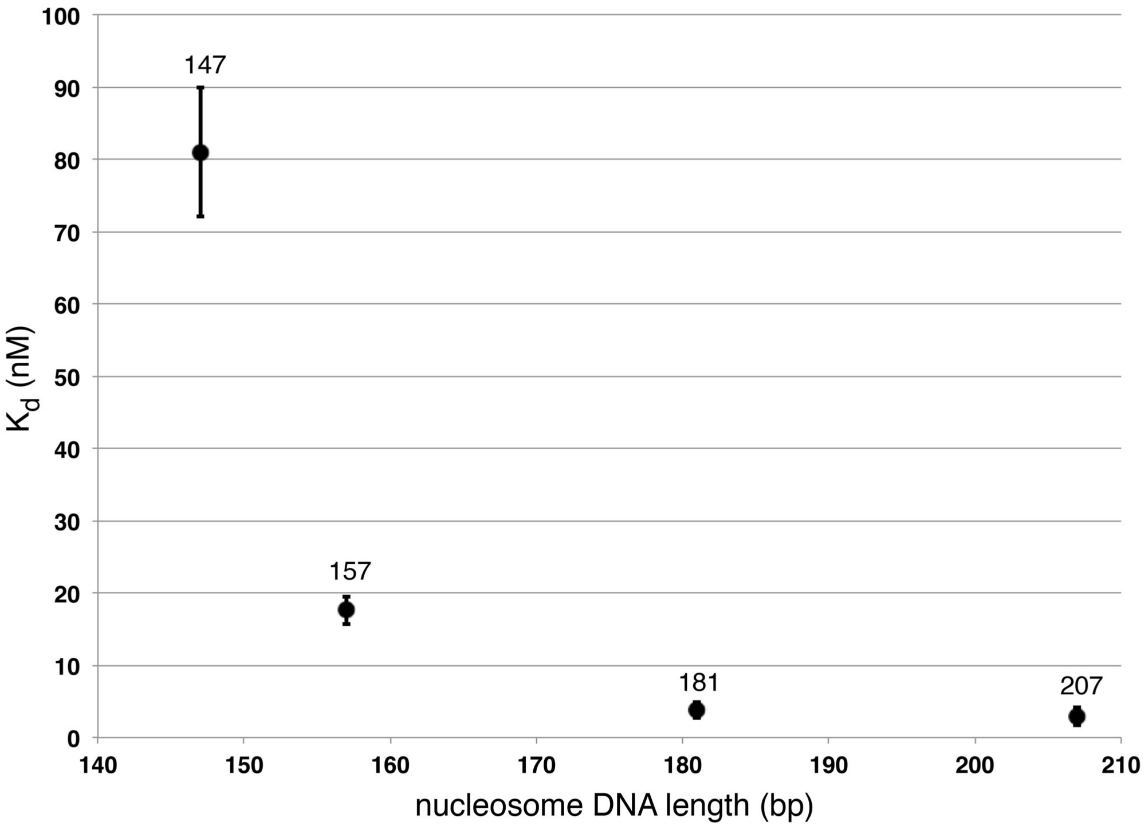

Supplementary Figure 2: Summary of dissociation constants for nucleosomes containing 147 bp , 157 bp, 181 bp and 207 bp symmetrically positioned DNA labeled on H3A21C. The standard deviations from three or more measurements are shown as vertical bars for each data point.
